# Piqures massives par un essaim d'abeilles chez un enfant

**DOI:** 10.4314/pamj.v10i0.72225

**Published:** 2011-10-10

**Authors:** Mohamed Adnane Berdai, Smael Labib, Monia El Balbal, Mustapha Harandou

**Affiliations:** 1 Service de Réanimation Mère et Enfant ; CHU Hassan II, Fès 30000, Maroc

**Keywords:** Abeille, envenimation, anaphylaxie, allergie, choc, Maroc

## Abstract

Les piqûres multiples d'abeilles sont responsables d'envenimation sévère. Nous rapportons un cas d'une attaque massive par un essaim d'abeilles chez un enfant de sept ans. Sa gravité est liée à la localisation céphalique et au nombre important des piqûres qui était d'environ 270. Ses complications étaient l'insuffisance rénale, l'anémie et une conjonctivite. La prise en charge était symptomatique avec bonne évolution clinique et biologique.

## Introduction

Les piqûres d'abeilles sont très fréquentes parfois mortelles. Il peut s'agir d'une piqûre simple responsable d'une réaction locale avec urticaire ou de piqûres multiples causant un syndrome toxique avec des signes systémiques. L'intensité de l'envenimation et le pronostic sont directement liés au nombre de piqûres d'abeilles.

## Patient et observation

**Figure 1:**
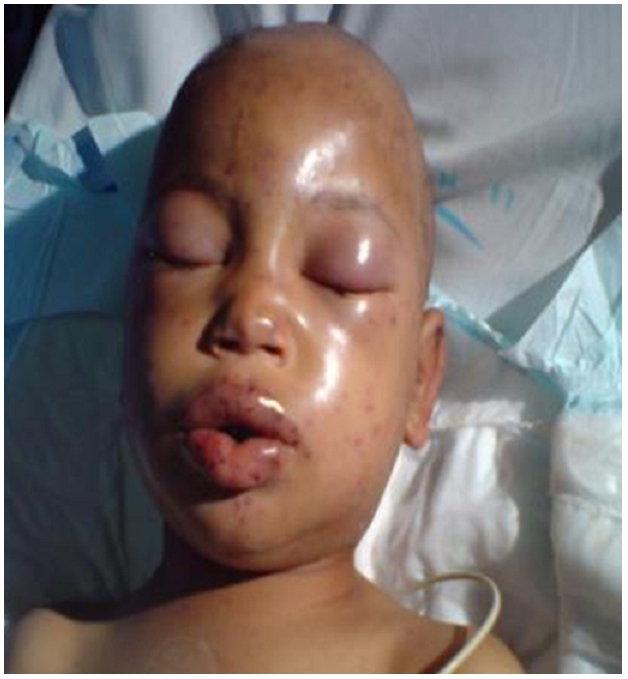
Localisation céphalique des piqûres avec œdème palpébral et des lèvres

Il s'agit d'un enfant de 7 ans, sans antécédents pathologiques notables, victime de piqûres par un essaim d'abeilles intéressant la tête, le visage, le cuir chevelu et les quatre membres occasionnant chez lui des douleurs atroces et des vomissements. Admis une heure trente minutes après l'attaque. L'examen a trouvé un enfant conscient hurlant de douleur, un œdème du cou et de la face comprenant les lèvres, les paupières avec occlusion oculaire complète et le cuir chevelu, apyrétique, tachycarde à 130 b/min, hypertendu à 150/70 mmHg (Figure 1).

On a noté la présence d'environ 270 piqûres avec persistance des dards au niveau du cuir chevelu, la face, les oreilles, le thorax et les extrémités des quatre membres.

L'enfant a bénéficié d'une oxygénothérapie, trois tests de remplissage par 20 cc/kg de sérum salé isotonique, analgésie par perfusion de paracétamol 15 mg/kg et nalbuphine à raison de 0,05 mg/kg/h, d'où le ralentissement de la fréquence cardiaque, corticothérapie à forte dose à base de méthylprednisolone 2 mg/kg/jour, antiémétiques et antihistaminiques. Par la suite on a procédé à l'ablation des dards avec désinfection cutanée et soins locaux biquotidiens. Le bilan biologique a révélé une hyperleucocytose à 37720 éléments par mm3 à prédominance polynucléaire neutrophile, des CPK-MB à deux fois la valeur normale et une fonction rénale normale initialement. La tomodensitométrie cérébro-orbitaire était normale.

**Figure 2:**
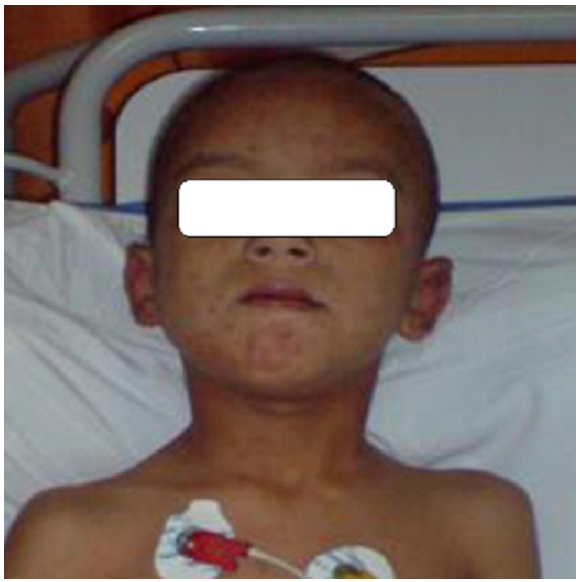
Amélioration clinique des lésions engendrées par les piqures d'abeilles

L’évolution a été marquée deux jours plus tard par l'apparition d'une conjonctivite d'où sa mise sous antibiothérapie locale (Tobramycine) et par l'installation une anémie hémolytique à 8,2 g/l bien tolérée et une insuffisance rénale (urée: 1,28 g/l, créatinine: 25 mg/l) à diurèse conservé suite à l'hyperhydratation. La fonction rénale s'est corrigée au huitième jour et La régression totale de l’œdème de face a été vers le dixième jour avec comme séquelle un ptosis persistant (Figure 2). La sortie à domicile est faite au 15e jour de son hospitalisation.

## Discussion

A l'inverse de la réaction anaphylactique immunologique, la toxicité de l'envenimation massive par les abeilles est le résultat de l'effet toxique direct de grandes quantités de venins injectés, le venin des abeilles est un mélange complexe d'enzymes, peptides et amines. La Mellitine, le composant induisant initialement la douleur, constitue 50% du poids du venin et agit en association avec la phospholipase A2 comme un agent cytolytique [1]. L'hyaluronidase augmente la perméabilité capillaire et facilite la diffusion des toxines [2].

Notre observation rapporte le cas d'une envenimation massive par un essaim d'abeilles dont la gravité réside dans le nombre de piqûres (environ 270), la localisation surtout céphalique et l’âge (7 ans). Sa sévérité vient de ses signes cliniques: l’œdème de la face et paracliniques: l'insuffisance rénale et l'anémie. On considère que le seuil létal, pour un adulte, se situe aux alentours de 400 piqures, mais des décès ont été rapportés à partir d'une trentaine de piqures simultanées [3], ce nombre devra être moins important chez l'enfant du fait d'un ratio poids corporel/venin diminué.

Les effets immédiats après les piqures massives sont des douleurs localisées, des sueurs, un érythème localisé au site de la piqure. Les signes systémiques précoces incluent asthénie, confusion, nausée, vomissements et diarrhée, dans les 24 heures peut se développer une hémolyse, une hémoglobinurie, une rhabdomyolyse ainsi qu'une cytolyse hépatique [4].les atteintes neuroencephaliques et cardiaques sont plus rares [5]. L'atteinte rénale souvent aigue est liée à la toxicité directe du venin sur les tubules, à l'hypovolémie prolongée ou à une tubulopathie secondaire à la myoglobinurie et l'hémoglobinurie, parfois elle survient à distance sous forme de syndrome néphrotique [3].

Dans notre observation, l’élévation minime des CPK-MB et l'hyperhydration précoce, indiquent que l'insuffisance rénale est probablement du à l'action direct du venin, elle est corrigée au 8e jour en assurant une hyperhydratation et une diurèse conservé. Bien que ces atteintes rénales guérissent habituellement sans séquelles [5], des cas d'insuffisance rénale ayant nécessité l'hémodialyse 48 heures après l'incident avec une récupération partielle de la fonction rénale ont été rapportées [6].

Le siège des piqûres a une valeur pronostique, la tête et le cou étant les zones les plus dangereuses, ainsi que les localisations pharyngo-laryngées qui peuvent causer le décès par l’œdème pharyngé et l'obstruction des voies respiratoires [7]. Les lésions oculaires par piqure d'abeille se manifeste par une douleur aigu, une injection conjonctivale, un chémosis et un œdème de la cornée [2], notre cas n'a pas présenté de lésions oculaires directes, il a par contre développé une conjonctivite qui a bien évolué sous antibiothérapie locale.

Etant donné que les manifestations cliniques initiales des piqures massives d'abeilles sont similaires aux réactions anaphylactiques aiguës, les mesures anti-anaphylactiques doivent donc être appliquées immédiatement, tels que le remplissage vasculaire, la corticothérapie et l’épinephrine en sous cutanée [8], La vasopermeabilité induite par le venin rendent ces mesures nécessaires pour maintenir une volémie efficace malgré une hypertension artérielle initiale due aux amines du venin [3,9]. Par ailleurs, Il faut maintenir un débit urinaire adéquat grâce à une hydratation agressive pour prévenir une insuffisance rénale induite par la rhabdomyolyse [4].

Les anomalies biologiques et les signes de toxicité peuvent manquer aux premières heures, ce qui rend impératif un monitorage prolongé avec une surveillance rapprochée de la biochimie sanguine, l'hospitalisation en unité de soins intensifs s'impose en cas de piqures d'abeilles dépassant 50 pour l'adulte et 1 piqure par kg pour l'enfant [4].

La prise en charge précoce est indispensable. Ainsi dans notre observation, l'admission précoce de l'enfant, le remplissage vasculaire massif, la corticothérapie à forte dose et l'ablation des dards étaient efficaces malgré l'attaque massive et la localisation dangereuse céphalique.

Le traitement local reste l'ablation des dards par un grattage léger en évitant toute pression qui viderait la glande cela a pour effet de réduire l'accès du venin à la circulation. Une désinfection locale par un antiseptique, de l'eau oxygénée ou de l'eau de javel doit être systématique [3,10]. L'analgésie par des morphinomimétiques et du paracétamol doit être préconisée comme dans notre cas.

Le pronostic de l'envenimation est corrélé aux concentrations plasmatiques du venin [11], De ce fait, dans les formes graves, une plasmaphérèse précoce serait utile, elle permettra d'une part de réduire les concentrations du venin circulant, et d'autre part l’épuration des médiateurs circulants suscités par le venin lui-même [8].

## Conclusion

Les envenimations massives par piqûres d'abeilles sont graves par leurs complications locales et systémiques pouvant parfois être mortelles. Le pronostic dépend du nombre de piqûres, de leur localisation et surtout du délai et de la qualité de prise en charge.
